# Tissue Factor and Extracellular Vesicles: Activation of Coagulation and Impact on Survival in Cancer

**DOI:** 10.3390/cancers13153839

**Published:** 2021-07-30

**Authors:** Yohei Hisada, Nigel Mackman

**Affiliations:** UNC Blood Research Center, Department of Medicine, University of North Carolina at Chapel Hill, Chapel Hill, NC 27599, USA; yohei_hisada@med.unc.edu

**Keywords:** animal model, cancer, disseminated intravascular coagulation, extracellular vesicle, survival, tissue factor, venous thrombosis

## Abstract

**Simple Summary:**

The tissue factor (TF)-factor VIIa complex is the major physiological initiator of blood coagulation. Tumors express TF and release TF-positive extracellular vesicles (EVs) into the circulation, and this is associated with the activation of coagulation. Circulating levels of EVTF activity may be a useful biomarker to identify patients at risk for thrombosis. Tumor TF and TF-positive EVs are also associated with reduced survival.

**Abstract:**

Tissue factor (TF) is a transmembrane glycoprotein that functions as a receptor for FVII/FVIIa and initiates the extrinsic coagulation pathway. Tumors and cancer cells express TF that can be released in the form of TF positive (TF+) extracellular vesicles (EVs). In this review, we summarize the studies of tumor TF and TF + EVs, and their association with activation of coagulation and survival in cancer patients. We also summarize the role of tumor-derived TF + EVs in venous thrombosis in mouse models. Levels of tumor TF and TF + EVs are associated with venous thromboembolism in pancreatic cancer patients. In addition, levels of EVTF activity are associated with disseminated intravascular coagulation in cancer patients. Furthermore, tumor-derived TF + EVs enhance venous thrombosis in mice. Tumor TF and TF + EVs are also associated with worse survival in cancer patients, particularly in pancreatic cancer patients. These studies indicate that EVTF activity could be used as a biomarker to identify pancreatic cancer patients at risk for venous thrombosis and cancer patients at risk for disseminated intravascular coagulation. EVTF activity may also be a useful prognostic biomarker in cancer patients.

## 1. Introduction

The risk of venous thromboembolism (VTE) is 4–9 times higher in patients with cancer compared with the general population [1,2]. Most of the VTE events are observed shortly before and after the diagnosis of cancer [3]. VTE is associated with increased mortality in cancer patients [4,5,6,7]. The risk of VTE in cancer patients has increased over recent years, which may be due to the increased survival of cancer patients, novel cancer therapies, and the increased diagnostic modalities [2]. There are several risk factors for cancer-associated VTE. These include tumor characteristics, treatment, and patients characteristics [8]. It is also notable that the incidence of VTE varies in patients with different types of cancer, with the highest incidence in pancreatic (~110/1000 person-years) and brain (~80/1000 person-years) cancer patients [9]. Therefore, it has been proposed that there are cancer-type specific pathways that enhance VTE [10]. Cancer patients also have disseminated intravascular coagulation (DIC) and a consumptive coagulopathy that results in bleeding. The frequency of bleeding varies with cancer type and is observed in 15–20% of patients with blood malignancy and ~7% of patients with solid tumors [11].

Tissue factor (TF) is a receptor for factor (F)VII/VIIa. The TF–FVIIa complex activates the extrinsic coagulation pathway [12]. TF plays an essential role in hemostasis [12]. Many types of cancers express TF [13]. Cancer cells release submicron membrane vesicles called extracellular vesicles (EVs) [14]. “Extracellular vesicle” is a collective term for any membrane vesicles released from cells, including microparticles, microvesicles, and exosomes [15]. TF expressing cancer cells release TF-positive EVs (TF + EVs).

This review summarizes studies of tumor TF and TF + EVs, and their association with VTE, DIC, and survival in cancer patients. In addition, we also summarize the role of tumor-derived TF + EVs in venous thrombosis in mouse models.

## 2. Tumor TF, VTE, and Survival in Cancer Patients

### 2.1. Tumor TF and Progression

Many studies have measured levels of tumor TF expression by immunohistochemistry. High levels of TF expression have been observed in different types of cancer. Pancreatic ductal adenocarcinoma (PDAC) expresses high levels of TF expression, whereas no TF expression was observed in normal pancreatic samples [16,17]. Higher levels of TF expression were observed in cervical carcinoma compared with adjacent normal tissue [18]. Importantly, TF expression increased with the progression of PDAC [16,19]. These data suggest that the level of TF expression is increased in tumors and is associated with tumor progression.

### 2.2. Tumor TF Expression and Tumor Gene Mutations

KRAS, tumor protein 53 (TP53), and serine/threonine kinase 11 (STK11) mutations are frequently detected in patients with lung cancer. In contrast, phosphatase and tensin homolog (PTEN) and anaplastic lymphoma kinase (ALK) gene mutation/rearrangement are less common [20,21,22,23]. Recent studies have found that some gene mutations are associated with increased TF expression. Mutation of KRAS, TP53, and PTEN in non-small cell lung carcinoma (NSCLC) was associated with high levels of TF mRNA expression [24,25]. Another study found significantly higher levels of TF protein expression in ALK-rearrangement positive NSCLC tumors compared to ALK-rearrangement negative NSCLC tumors [26]. Recently, Dunbar and colleagues found that mutation of STK11 was associated with increased TF expression in patients with lung adenocarcinoma [27]. Mutations in isocitrate dehydrogenase 1 or 2 (IDH1/2) are frequently detected in patients with glioma. Unruh and colleagues found that mutations of IDH1/2 are associated with increased methylation of the *F3* (TF gene) promoter and decreased TF expression in glioblastoma multiforme (GBM) [28]. These data indicate that gene mutations are associated with increased and decreased TF expression.

Mouse studies have shown that the formation of the TF-FVIIa complex on the surface of tumor cells promotes tumor growth by activating protease-activated receptor (PAR) 2 signaling and by increasing vascular endothelial growth factor expression [29,30,31]. In addition, tumor TF enhances metastasis in mice [32,33]. These studies indicate that tumor TF contributes to tumor growth and metastasis.

### 2.3. Tumor TF and VTE in Cancer Patients

Khorana and colleagues found that pancreatic cancer patients with high TF expression in tumors had a high rate of symptomatic VTE (26.3%) compared to a low rate (4.5%) in pancreatic cancer patients with low TF expression in their tumors [17]. However, Thaler and colleagues found no association between tumor TF expression and VTE in brain cancer patients [34]. These data suggest that TF expression in pancreatic cancer plays a role in VTE but not brain cancer.

### 2.4. Tumor TF and Survival in Cancer Patients

The association between tumor TF expression and survival has been analyzed in many types of cancer, including pancreatic [19], colorectal [35], gastric [36], esophageal [37], breast [38], prostate [39], and bladder [40] cancer. Nitori and colleagues found that high tumor TF expression was associated with decreased survival in patients with PDAC [19]. Tumor TF expression was also a predictor of survival in patients with metastatic prostate cancer [39]. Another group reported that the 3-year survival after cancer diagnosis was 88% in colorectal cancer patients with TF-negative tumors and 39% in colorectal cancer patients with TF-positive tumors [35]. Similarly, high tumor TF expression was associated with worse survival in patients with esophageal squamous cell carcinoma [37]. Patry and colleagues reported a 3.15-fold increased risk of cancer-specific death in bladder cancer patients with TF-positive tumors compared to bladder cancer patients with TF-negative tumors [40]. Interestingly, in gastric cancer patients, high tumor TF expression was associated with worse survival in patients with intestinal-type carcinoma but not in patients with diffuse-type carcinoma [36]. Inconsistent results have been reported for tumor TF expression and survival in breast cancer patients. Ueno and colleagues found that increased tumor TF expression was associated with decreased survival in breast cancer patients [38]. In contrast, Stampfli and colleagues found that there was no association between tumor TF and survival in breast cancer patients [41]. These studies suggest that tumor TF expression is a marker of cancer prognosis.

### 2.5. Targeting Tumor TF to Kill Tumors

TF has been used as a target to kill tumor cells. One strategy was to induce infarction in the tumor vasculature using an antibody that targets tumor vascular endothelium linked to truncated TF [42,43,44,45]. This form of TF has low coagulant activity in the circulation but is prothrombotic when localized to the tumor vasculature [42]. This strategy reduced tumor growth in mouse models but was not used clinically, possibly due to the risk of thrombosis. The second series of studies used an immunoconjugate of the Fc region of a human IgG and mutated FVII, called ICON, to target TF expressed by tumors and the tumor endothelium [46,47,48]. Importantly, the active site of FVII is mutated so that the ICON does not induce coagulation. The tumor-killing mechanism of the ICON is due to the induction of natural killer cells by the Fc effector domain of human IgG. ICON was encoded in an adenoviral vector that was administered to the mice. This strategy also reduced tumor growth in mice [49,50]. In addition, it was evaluated in the phase 1 trial in patients with uveal melanoma and choroid neoplasm (ClinicalTrials.gov identifier: NCT02771340). The last series of studies evaluated the efficacy of anti-TF antibody-drug conjugate (ADC) in tumor growth in mice [51,52,53,54,55]. Among them, Tistotumab vedotin, which is a complex of an anti-TF antibody and a cytotoxic drug called monomethyl auristatin E (MMAE), is the most promising ADC. There are nine clinical trials using Tisotumab vodotin (ClinicalTrials.gov identifier: NCT03245736, NCT03485209, NCT03438396, NCT03657043, NCT02552121, NCT02001623, NCT03913741, NCT03786081, and NCT04697628) [56]. Another anti-TF antibody-MMAE conjugate called XB002 will also be evaluated in a clinical trial in the near future (ClinicalTrials.gov identifier: NCT04925284). These studies indicate that targeting tumor TF is a promising approach to treat solid tumors.

## 3. TF-Positive Extracellular Vesicles in Cancer Patients

### 3.1. Measurement of TF + EVs in Plasma

Dvorak and colleagues were the first to report that cancer cells release membrane vesicles that have procoagulant activity [57]. Since then, several studies have shown that these membrane vesicles carry TF that accounts for their procoagulant activity [58,59,60].

In general, there are two ways of measuring levels of TF + EVs in plasma: TF antigen-based assays and TF activity-based assays. TF antigen assays include ELISA and flow cytometry, whereas TF activity assays include activated FX (FXa) generation assays and clotting assays. Previous studies showed that TF activity assays have higher sensitivity and specificity than TF antigen assays [61,62,63]. A disadvantage of TF antigen assays is that they detect both active and inactive forms of TF, whereas TF activity assays only detect active TF. TF antigen assays usually use plasma, whereas TF activity assays use isolated EVs. The TF signal is amplified in the activity assays, making them more sensitive than the antigen assays. In addition, it should be noted that some commercial TF ELISAs do not accurately measure levels of TF antigen in plasma [61,64,65,66].

We and others developed in-house EVTF activity assays [63,67,68]. In brief, EVs are isolated from plasma samples using high-speed centrifugation and washed to remove plasma proteins. Isolated EVs are incubated with FVIIa and FX to generate FXa. Finally, FXa generation is measured using an FXa-specific chromogenic substrate. The assay is performed with either an anti-TF antibody or control antibody to distinguish TF-dependent versus TF-independent FXa generation. The two in-house EVTF activity assays are similar, and one study showed a significant correlation between the two assays [69]. Importantly, we found that our in-house EVTF activity assay had a higher sensitivity and specificity compared with a commercially available EVTF activity assay called Zymuphen^TM^ MP-TF [70]. The lower sensitivity of Zymuphen^TM^ MP-TF is likely due to the reduced recovery of EVs compared with centrifugation. The lower specificity may be due to the use of a high concentration of FVIIa in the assay. Another study measured EVTF activity in a clotting assay [63].

### 3.2. Association between EVTF Activity and VTE in Cancer Patients

EVTF activity was found to be increased in cancer patients with VTE [68,71,72]. However, a complication with these studies is that the VTE itself may contribute to the increase in levels of EVTF activity. Prospective studies have analyzed the association between levels of EVTF activity and VTE in patients with different types of cancer. Several studies showed that there was a significant association between EVTF activity and VTE in patients with pancreatic cancer [73,74,75]. A borderline non-significant association was reported between EVTF activity and VTE in patients with pancreatic cancer but not in patients with brain, stomach, and colorectal cancer [69]. However, the follow-up term of this study was 2 years, which is longer than other studies that had follow-up times of 5–6 months. Another study reported an association between EVTF activity and VTE in patients with different types of cancer, although this association appeared to be due to PDAC patients in the population [76]. Glioma patients with wild-type *IDH1/2* have a higher rate of VTE than patients with mutant *IDH1/2*. Interestingly, Unruh and colleagues found higher levels of EVTF activity in glioma patients with wild-type *IDH1/2* compared to glioma patients with mutant *IDH1/2* [28]. Other studies found that there was no association between EVTF activity and VTE in patients with small cell lung carcinoma [77], multiple myeloma [78], and ovarian cancer [79,80].

Several longitudinal studies have analyzed the association between EVTF activity and VTE in cancer patients. Our early study measured EVTF activity in 10 pancreatic cancer patients, 2 of whom had a VTE [67]. Interestingly, there was a stepwise increase in EVTF activity in the two VTE patients, whereas there are no significant changes over time in the patients without VTE. More recently, we measured EVTF activity in 13 pancreatic cancer patients, 1 of which had a VTE, and 22 colorectal cancer patients, 4 of which had a VTE [81]. The pancreatic cancer patient with a VTE had a stepwise increase in EVTF activity prior to the VTE. In contrast, none of the colorectal cancer patients with VTE had increased levels of EVTF activity prior to the VTEs. One recent study did not find an association between EVTF activity and VTE in patients with four different cancer types, including pancreatic cancer [82]. However, this study did not perform an analysis of the individual cancer types because of the small number of VTE events. Table 1 summarizes studies evaluating the association between EVTF activity and VTE in patients with cancer (Table 1).

These data suggest that there is an association between EVTF activity and VTE in pancreatic cancer but not in other types of cancer.

### 3.3. Association between TF Antigen and EVTF Activity and DIC in Cancer Patients

TF has been proposed to be involved in DIC in cancer patients due to its strong procoagulant activity [83]. Early studies measured plasma TF antigen levels. One study found significantly increased levels of TF antigen in leukemia patients with DIC compared to leukemia patients without DIC [84]. Similarly, another study detected high levels of TF antigen in 46.2% of patients with DIC, most of whom had solid tumors and acute leukemia [85]. Asakura and colleagues found increased TF antigen levels in DIC patients with solid tumors but did not observe increased TF antigen levels in DIC patients with different types of leukemia and non-Hodgkin lymphoma [86]. However, as noted above, the reliability of measuring TF antigen in plasma using some commercial ELISAs is questionable [65,66].

More recently, some small studies investigated the levels of EVTF activity in cancer patients with and without DIC. One study reported increased levels of EVTF activity in five cancer patients with DIC, including four with solid tumors and one with acute promyelocytic leukemia (APL) compared with healthy controls [63]. Interestingly, levels of EVTF activity were increased 6–193-fold compared to the mean in healthy controls. Levels of plasma TF antigen were also increased but to a lesser extent than EVTF activity (2–10-fold). Another group found that one APL patient and one acute myelocytic leukemia (AML) patient with DIC had high levels of EVTF activity compared to an AML patient without DIC [87]. Interestingly, EVTF activity levels of the two DIC patients became undetectable after the cessation of DIC. More recently, the same group observed significantly increased levels of EVTF activity in seven prostate cancer patients with DIC compared with ten prostate cancer patients without DIC [88]. EVTF activity was between 24 and 150-fold higher in the patients with DIC compared to healthy controls. These studies demonstrate an association between high levels of EVTF activity and DIC in cancer patients.

### 3.4. Association between EVTF Activity and Survival in Cancer Patients

Similar to tumor TF, EVTF activity is also associated with survival in cancer patients. Several studies using multiple cancer types have shown an association between EVTF activity and survival [68,71,89,90]. Other studies with only pancreatic cancer patients also found an association between EVTF activity and survival [69,73,74,91]. These results are consistent with the observation that TF expression is increased with cancer progression and that EVTF activity could be used as a prognostic marker for survival. Table 2 summarizes studies evaluating the association between EVTF activity and survival in patients with cancer (Table 2).

## 4. Role of TF-Positive Extracellular Vesicles in Mice

### 4.1. Choice of Mouse Models

We and others have used mouse models to investigate the role of tumor-derived TF + EVs in thrombosis. There are several factors to consider when selecting a suitable mouse model [92]. The first choice is the species. Immunocompetent mice, such as C57BL/6 and BALB/c mice, are used for allograft models, whereas immunodeficient mice, such as nude mice or severe combined immunodeficient (SCID) mice, are used for xenograft models. The second choice is the type of cancer cell. There are many human cancer cell lines and patient-derived xenografts available, but the number of mouse cancer cells is limited. The third choice is the tumor site. Subcutaneous tumor or orthotopic tumor models are commonly used. Orthotopic tumors are more clinically relevant models, but tumor insertion requires survival surgery, and measurement of tumor size requires labeling of the tumor cells and in vivo imaging. The final choice is the thrombosis model. The inferior vena cava (IVC) stenosis model and stasis models are used to evaluate venous thrombosis in mice [93]. Alternatively, ferric chloride can be used to induce thrombosis [93].

### 4.2. Studies of TF + EVs in Tumor Bearing Mice

Several studies with mice bearing human colorectal tumors, pancreatic tumors, or brain tumors have shown that tumors release human TF + EVs into the circulation [31,94,95,96,97,98]. Plasma human TF antigen levels correlated with tumor size [31,94]. In addition, there was a correlation between plasma human TF antigen levels and levels of thrombin–antithrombin (TAT) complex, which is a marker of activation of coagulation. Another study found that C57BL/6 mice bearing murine pancreatic Panc02 tumors expressing GFP release GFP + EVs into the circulation, and these accumulate at the site of laser injury in venules [99]. These studies suggest that circulating TF+ EVs contribute to the activation of the coagulation system in tumor-bearing mice.

### 4.3. Injection of TF + EVs Increases Thrombosis

Several studies have shown that injection of TF + EVs into mice increases thrombosis [95,96,100,101,102]. Importantly, treatment of human tumor-derived EVs with an anti-human TF antibody significantly reduced the incidence and weight of venous thrombosis in C57BL/6 mice [96,100]. Another group found that the incidence of thrombosis was significantly reduced in mice injected with EVs from TF-knockdown Panc02 cells compared with injection of EVs from TF-expressing Panc02 cells [101]. More recently, Sasano and colleagues showed that injection of EVs from an ovarian A2780 cell line overexpressing human TF significantly increased thrombus weight in nude mice compared with injection of EVs from native A2780 cells [102]. These data indicate that injection of tumor-derived TF+ EVs enhances venous thrombosis in mice.

### 4.4. Tumor-Derived TF + EVs Increase Thrombosis

The use of mice bearing tumors is a more physiologic model compared to the injection of TF + EVs into mice without tumors. An early study found that Panc02 tumor-bearing mice and murine lung LLC tumor-bearing mice significantly shortened time to occlusion in ferric chloride-induced mesenteric arterioles and venules occlusion models [99]. Similarly, we found that mice bearing human pancreatic HPAF-II tumors had a significantly shortened time-to-occlusion in a ferric chloride-induced saphenous vein thrombosis model compared with mice without tumors [95]. These data indicate mice-bearing tumors have enhanced ferric chloride-induced thrombosis.

Mixed results were observed in the IVC stenosis model. One study found that mice bearing Panc02 tumors had increased thrombus weight compared to control mice [101]. In contrast, we found that the mice bearing human pancreatic tumors (HPAF-II and BxPC-3) did not change thrombus weight and incidence of thrombosis compared to control mice [95,96]. This difference may be due to the use of immunocompetent versus immunodeficient mice. In contrast to our negative results with the IVC stenosis model, we found that mice bearing large human pancreatic tumors had significantly larger thrombi in an IVC stasis model compared with thrombi in mice without tumors [97]. Importantly, treatment of an inhibitory anti-human TF antibody that does not cross-react with mouse TF significantly reduced thrombus size in BxPC-3 tumor-bearing mice but not in control mice [97]. These data indicate that TF + EVs in circulation enhance venous thrombosis in tumor-bearing mice.

### 4.5. Pro-angiogenic and Pro-inflammatory Functions of TF + EVs

A few studies have investigated the non-hemostatic functions of TF + EVs. Svensson and colleagues found that TF + EVs derived from the human glioblastoma cell line U87-MG enhance the expression of pro-angiogenic, heparin-binding EGF via activation of the PAR2-ERK1/2 pathway in hypoxic human umbilical vein endothelial cells [103]. More recently, it was shown that TF + EVs derived from the human pancreatic cancer cell lines Capan-1 and BxPC-3 and the human breast cancer cell line MDA-MB-231 increase IL-8 and E-selectin expression in human umbilical vein endothelial cells [104]. These data indicate that TF + EVs have pro-angiogenic and pro-inflammatory functions by altering the characteristics of endothelial cells.

## 5. Conclusions

**Figure 1:**
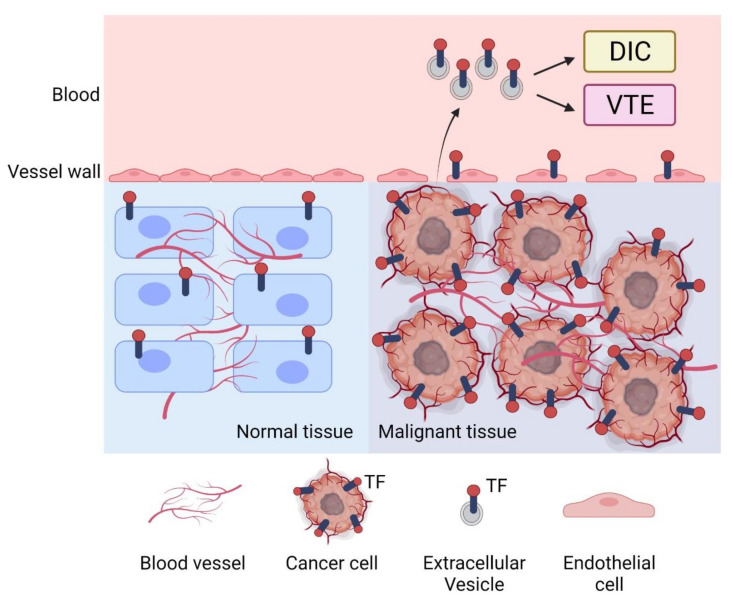
Tissue factor and cancer. Tissue factor (TF) contributes to tumor growth and angiogenesis in malignant tissue. Tumor cells release TF-positive extracellular vesicles that can cause either venous thromboembolism (VTE) or disseminated intravascular coagulation (DIC) in cancer patients. Figure is created with BioRender.com.

TF expression increases with tumor progression, and tumor cells release TF+ EVs (Figure 1). Levels of EVTF activity are associated with VTE in pancreatic cancer patients and DIC in cancer patients (Figure 1). In addition, levels of EVTF activity are associated with survival in cancer patients, particularly in pancreatic cancer patients. Furthermore, tumor-derived TF + EVs enhance venous thrombosis in mice. EVTF activity may be a good biomarker to identify the risk of VTE and DIC in cancer patients. EVTF activity may also be a prognostic biomarker in cancer patients.

## Figures and Tables

**Table 1:** Studies evaluating the association between extracellular vesicle tissue factor activity and venous thromboembolism in patients with cancer.

Study	Tumor Type	No. of Patients with VTE/Total No. of Patients	% of VTE	Association between EVTF Activity and VTE
Single time point studies				
van Doormaal et al. [76]	More than 6 different types of cancer	5/43	11.6	Yes *
van Es et al. [75]	9 different types of cancer	40/648	6.2	Yes **
Bharthuar et al. [73]	Pancreaticobiliary	52/117	44.4	Yes
Woei A-Jin et al. [74]	Pancreatic	14/79	17.7	Yes
Thaler et al. [69]	Pancreatic	12/60	20	No
	Brain	6/43	14	No
	Colorectal	12/126	9.5	No
	Stomach	19/119	16	No
Gezelius et al. [77]	Small cell lung carcinoma	15/235	6.3	No
Cohen et al. [79]	Epithelial ovarian	19/59	32.2	No
Hisada et al. [80]	Ovarian	4/84	4.8	No
Auwerda et al. [78]	Multiple myeloma	15/122	12.3	No
Longitudinal studies				
Khorana et al. [67]	Pancreatic	2/10	20	2 patients with serial increases in EVTF activity had VTE
Kasthuri et al. [81]	Pancreatic	1/13	7.7	1 patient with increased EVTF activity had VTE
	Colorectal	4/22	18.2	All VTE patients did not have increased EVTF activity
Reitter et al. [82]	4 different types of cancer	12/38	31.6	No

EVTF, extracellular vesicle tissue factor; VTE, venous thromboembolism; * including 3 pancreatic cancer patients with VTE; ** fibrin generation assay.

**Table 2:** Studies evaluating the association between extracellular vesicle tissue factor activity and survival in patients with cancer.

Study	Tumor Type	Total No. of Patients	Association between EVTF and Mortality
Tesselaar et al. [68]	Pancreatic	23	Yes
Tesselaar et al. [71]	13 different types of cancer	100	Yes
Thaler et al. [69]	Pancreatic	60	Yes
	Brain	43	No
	Colorectal	126	No
	Stomach	119	Yes *
Thaler et al. [91]	Pancreatic	73	Yes
Bharthuar et al. [73]	Pancreaticobiliary	117	Yes
Hernandez et al. [89]	Stomach	25	Yes **
	Colorectal	96	
	Pancreatic	9	
	Lung	18	
	Breast	42	
	Non-Hodgkin lymphoma	62	
Woei et al. [74]	Pancreatic	79	Yes
Hisada et al. [90]	17 different types of cancer	60	Yes

EVTF, extracellular vesicle tissue factor; * multivariable analysis; ** Yes for all the cancers combined.
